# Absence of Stress Response in Dorsal Raphe Nucleus in Modulator of Apoptosis 1-Deficient Mice

**DOI:** 10.1007/s12035-018-1205-7

**Published:** 2018-07-12

**Authors:** Hui Zhao, Nur-Ezan Mohamed, Su Jing Chan, Chong Teik Tan, Ran Tao, Victor C. Yu, Peter T.-H. Wong

**Affiliations:** 1 Department of Pharmacology, Yong Loo Lin School of Medicine, National University of Singapore, 16, Medical Drive, #04-01, Singapore, 117600 Singapore; 2 Department of Radiology, Massachusetts General Hospital, Harvard Medical School, Charlestown, MA 02129 USA; 3 Institute of Medical Biology, Glycotherapeutics Group, 8A Biomedical Grove, #06-06 Immunos, A*STAR, Singapore, 138648 Singapore; 4 Department of Pharmacy, Faculty of Science, National University of Singapore, Singapore, Singapore

**Keywords:** Modulator of apoptosis, Stress, Depression, Serotonin, Dorsal raphe nucleus, Brain-derived neurotrophic factor

## Abstract

Modulator of apoptosis 1 (MOAP-1) is a Bcl-2-associated X Protein (BAX)-associating protein that plays an important role in regulating apoptosis. It is highly enriched in the brain but its function in this organ remains unknown. Studies on BAX^−/−^ mice suggested that disruption of programmed cell death may lead to abnormal emotional states. We thus hypothesize that MOAP-1^−/−^ mice may also display stress-related behavioral differences and perhaps involved in stress responses in the brain and investigated if a depression-like trait exists in MOAP-1^−/−^ mice, and if so, whether it is age related, and how it relates to central serotonergic stress response in the dorsal raphe nucleus. Young MOAP-1^−/−^ mice exhibit depression-like behavior, in the form of increased immobility time when compared to age-matched wild-type mice in the forced swimming test, which is abolished by acute treatment of fluoxetine. This is supported by data from the tail suspension and sucrose preference tests. Repeated forced swimming stress causes an up-regulation of tryptophan hydroxylase 2 (TPH2) and a down-regulation of brain-derived neurotrophic factor (BDNF) in the dorsal raphe nucleus (DRN) in young wild-type (WT) control mice. In contrast, TPH2 up-regulation was not observed in aged WT mice. Interestingly, such a stress response appears absent in both young and aged MOAP-1^−/−^ mice. Aged MOAP-1^−/−^ and WT mice also have similar immobility times on the forced swimming test. These data suggest that MOAP-1 is required in the regulation of stress response in the DRN. Crosstalk between BDNF and 5-HT appears to play an important role in this stress response.

**Electronic supplementary material:**

The online version of this article (10.1007/s12035-018-1205-7) contains supplementary material, which is available to authorized users.

## Introduction

Modulator of apoptosis 1 (MOAP-1), a BAX-associating BH-3 like protein, is a short-lived protein enriched in the outer membrane of mitochondria [1, 2]. MOAP-1 plays an important role in regulating apoptosis by interacting with BAX [2, 3]. In response to a death stimulus, tumor necrosis factor (TNF)-α binds to its receptor TNF-R1 and causes it to internalize. The internalized TNF-R1 complexes with MOAP-1 and Ras association domain family 1A (RASSF1A), and triggers a conformational change in MOAP-1 to expose its BH3-like domain for BAX association. BAX then becomes activated and translocates to the mitochondrial outer membrane to initiate apoptosis by releasing cytochrome c and apoptogenic factors. It is suggested that MOAP-1, like RASSF1A, is itself a tumor suppressor protein [4]. MOAP-1 also mediates Fas-induced apoptosis in the liver and MOAP-1^−/−^ mice are protected from Fas apoptotic signaling-induced acute liver injury [5]. However, there is no reported unusual/abnormal phenotype in MOAP-1^−/−^ mice. It has been noted that these mice develop normally to adulthood and are fertile [5]. In our hands, they do not appear to differ significantly from MOAP-1^+/+^ mice in appearance, body weight (Fig. 5a), and fertility (litter size 6.3 ± 1.1 vs 6.1 ± 0.6 of WT (*n* = 4), 4 litters in 6 months for both). While we had not checked the natural lifespan of these MOAP-1^−/−^ mice, the mean ages of our aged mice used in this study were 23.5 ± 0.4 and 23.9 ± 0.4 months (range 22–26 months) for MOAP-1^−/−^ and MOAP-1^+/+^ mice, respectively, which is approximately 90% of the reported average lifespan of C57/BL6 mice [6, 7].

MOAP-1 is highly enriched in the mammalian brain with a ubiquitous presence in all brain regions while higher expression is seen in the cortex and cerebellum [unpublished data, 3, 8], but its role in this important organ is largely obscure. The abundance of MOAP-1 in the normal adult brain raises the question whether or not it plays any role in brain functions [8]. Interestingly, it has been reported that BAX^−/−^ mice exhibit a substantial increase in the number of sympathetic and motor neurons in the superior cervical ganglia and facial nuclei, respectively [9]. Increases in neuron numbers were also reported in hippocampal dentate gyrus [10]. More recently, behavioral phenotypes of BAX^−/−^ mice have emerged. Luedke et al. [11] reported reduced anxiety based on elevated plus maze (EPM) performance and different defensive profile in response to aversive odors when compared to wild-type control mice. Krahe et al. [12] reported increased total locomotor activities in the open field test (OFT) without changes in activity in the center zone. They suggested that impaired hippocampal and cerebellar function might underlie this apparent hyperactivity. Increased immobility time in the forced swimming test (FST) was also reported in these BAX^−/−^ mice, which was interpreted as a “freezing state” triggered by the exposure to a novel and stressful environment. These seem to contradict the earlier findings of increased hippocampal neurogenesis and reduced anxiety on the EPM [10, 11]. More recently, stronger evidence that relates BAX and stress was provided by Culig et al. [13]. Using iBAX mice in which BAX can be inducibly ablated by tamoxifen, these authors found that BAX ablation reversed the unpredictable chronic mild stress (UCMS)-induced elevated corticosterone levels and reduced hippocampal neurogenesis, while it had no effects on non-stressed controls. Behaviorally, the time spent in the dark box was decreased in the light dark box (LDB) test, but locomotor activity and sucrose preference were not changed.

The central serotonergic systems play an important role in the regulation of many types of behavior such as depression [14], aggression [15], and feeding [16], as well as in the regulation of stress response by controlling the hypothalamus-pituitary-adrenal axis (HPA) activity and glucocorticoid levels [17]. Serotonin or 5-hydroxytryptamine (5-HT) is probably the most studied neurotransmitter in relation to depression and the serotonin reuptake inhibitors (SSRI) remain as first-line drugs in pharmacotherapy for major depressive disorder. Upon administration of fluoxetine (Flx), extracellular levels of 5-HT increase rapidly in the brain [18] but therapeutic efficacy is seen only after a period of 2–3 weeks indicating an additional requirement of mechanisms that appear only after chronic administration. Several potential contributing factors have been proposed including hippocampal neurogenesis [19] and neurotrophic factors such as brain-derived neurotrophic factor (BDNF) [20]. Both 5-HT and BDNF positively modulate hippocampal neurogenesis, which then regulate stress response through the HPA [21]. Stress has been shown to decrease hippocampal BDNF, which is increased by chronic antidepressant treatment [22]. BDNF signaling is required for antidepressant efficacy and BDNF ablation causes behavioral changes and learning deficits [23].

With this background, we thus hypothesize, by association, that MOAP-1^−/−^ mice, like BAX^−/−^ mice, may display stress-related behavioral differences and perhaps involved in stress responses in the brain. Stress responsiveness is also known to be plastic and may vary within individuals in an adaptive manner and this within-individual plasticity may explain age-related decrease in stress response [24, 25]. Therefore, we are interested to investigate if MOAP-1 plays a role in behavioral responses in a number of different acute stress paradigms using MOAP-1^−/−^ mice and, if so, whether or not it is age related. Significantly, how MOAP-1 would be involved in the control of the serotonergic stress response in the dorsal raphe nucleus (DRN). In this connection, early work has shown that acute and chronic stress could decrease BDNF in the hippocampus [26, 27] and BDNF is known to exert influence on serotonergic transmission in the hippocampus [28] and amygdala [29]. However, little is known about the relationship between 5-HT and BDNF in the DRN. Therefore, we also investigated any potential interplay between BDNF and the serotonergic system in this nucleus.

## Materials and Methods

### Animals and Treatment

All animal procedures were carried out with approval and oversight from the Institutional Animal Care and Use Committee (IACUC) of the National University of Singapore and conducted in compliance with the National Advisory Committee for Laboratory Animal Research (NACLAR) guidelines. MOAP-1^−/−^ mice were developed as previously described [5]. Briefly, the targeting vector was designed to replace the entire coding region of MOAP-1 exon 2 gene with the neomycin cassette via homologous recombination. The linearized targeting vector was introduced into murine embryonic stem cells via electroporation. Heterozygous mice were backcrossed with the C57/BL6 strain for more than ten generations to generate homozygous MOAP-1^−/−^ mice. Genotypes of the mice were confirmed by PCR and Western blot. Both MOAP-1^+/+^ and MOAP-1^−/−^ mice used for experiments were from the same heterozygous founders in pure C57/BL6 genetic background and were in-bred for no more than six generations.

Mice were maintained on a 12:12 h light/dark cycle with standard chow and water. Young (3–6 months old) and aged (22–26 months old) mice were used in this study. Flx hydrochloride (Sigma-Aldrich, Saint Louis, MO, USA, 10 or 20 mg/kg, 10 ml/kg) was administered by intraperitoneal injection 30 min before behavioral testing [30]. Control mice received the vehicle normal saline.

### Behavioral Tests

All behavioral tests were carried out between 1000 and 1700 h. Mice were acclimatized to the experimental room for at least 1 h before testing. Young mice were divided into four groups and each group was subjected to the following behavioral tests: (1) Rotarod and LDB; (2) OFT and EPM; (3) FST and TST; and (4) sucrose preference test (SPT). Only one group of aged mice were used for Rotarod, FST, and TST. Behavioral tests on the same animals were separated by at least 2 weeks. The experimenter was always blinded to the genotype of the mice.

#### Forced Swimming Test

Forced swimming test (FST) was carried out as previously described [31]. Young (*n* = 15–17) and aged (*n* = 14–15) mice were individually placed inside a transparent glass cylinders (20 cm diameter × 40 cm high) filled with water (25 ± 2 °C) to a depth of 20 cm. The mouse was placed in the water for 6 min and the immobility time (time spent motionless floating) and swimming time (time spent in large horizontal movements) were recorded over the last 4 min.

#### Tail Suspension Test

Tail suspension test (TST) was performed in accordance with the original method [32]. Young (*n* = 15–18) and aged (*n* = 14) mice were suspended in mid-air by its tail on a horizontal rod 50 cm above ground. The total immobility time, defined as time spent hanging motionless, was recorded in a 5-min trial session. Dalvi and Lucki [33] noted that C57 mice were inappropriate for the TST because of a tendency of these mice to grasp their tails with their front paws and climb up to the horizontal bar. However, we had not observed such behavior in our mice. This may be attributable to the fact that our mice were produced through heterozygotic breeding pairs, which was recommended to circumvent such confounding factors [34].

#### Rotarod Test

The rotarod apparatus (Ugo Basile, Varese, Italy) was used to assess motor function. Young (*n* = 14) and aged (*n* = 15–17) mice were placed on a rotating rod that accelerates from 5 to 20 rpm in a 5-min period. The time the animal remained on the rod before falling down was recorded. Each mouse was subjected to three trials at 10-min interval. Mice that do not fall after 5 min will be removed and assigned a time of 5 min. The average time recorded for the second and third trials were used for data analysis.

#### Elevated Plus Maze

The apparatus consists of four arms (30 cm long) in the shape of a + sign elevated 50 cm above the floor. Two closed arms opposite each other are enclosed with 20 cm high walls and the remaining two open arms have no walls. For the elevated plus maze (EPM) test, a mouse (*n* = 10) was initially placed at the center facing a closed arm and allowed to explore the arms for 5 min. The duration spent in each arm and the total arm entries were recorded using Ethovision XT software (Noldus Information Technologies, Wageningen, Netherlands).

#### Open Field Test

The apparatus consist of a circular tank (120 cm diameter, 50 cm deep) divided into three zones: peripheral, middle, and center zone. The tank was illuminated with red light with no other illumination in the room. Mice (*n* = 10–12) were individually placed in the peripheral zone facing outwards and left to explore for 10 min. The time and distance spent in zones were monitored by video-tracking system and quantified by Ethovision XT software (Noldus Information Technologies, Wageningen, Netherlands).

#### Light Dark Box

The box consists two compartments, one is covered and dark while the other is open and brightly-lit. Mice (*n* = 15) were placed in the bright compartment facing the dark compartment and left to explore for 5 min. The time spent in each compartment was monitored by Ethovision XT software (Noldus Information Technologies Wageningen, Netherlands).

#### Sucrose Preference Test

Mice (*n* = 8) were individually housed in their home cages with two bottles (one containing 2% sucrose solution and the other containing water) for 7 days to habituate to these test conditions (adaptation period). The stressed group was subjected to repeated forced swimming for 15 min daily from day 5 to 7. After the adaptation period, the sucrose preference test was conducted for 4 days. Sucrose solution and water consumption were measured daily by weighing the bottles and the position of bottles were reversed daily to prevent the potential effect of location preference. Sucrose preference (%) was calculated as sucrose intake (g) / [sucrose intake (g) + water intake (g)] × 100%.

### Repeated Forced Swimming Stress and Tissue Collection

Mice were subjected to repeated forced swimming as described in FST for 15 min daily for 3 days (3d-RFSS). Mice were anesthetized with ketamine/medetomidine 1 h after the last swim stress exposure. Blood samples were collected by cardiac puncture. Plasma was separated from whole blood by centrifugation and stored at – 80 °C until use. Brains were perfused and fixed for immunofluorescence staining or collected and frozen immediately in liquid nitrogen and stored at – 80 °C until use.

### Western Blotting

Brain tissues from MOAP-1^−/−^ and WT mice were homogenized in RIPA buffer (Cell Signaling Technologies, Danvers, MA, USA) with a phosphatase and protease inhibitor cocktail (Roche, Mannheim, Germany). Proteins were run on 10% SDS/PAGE gel, transferred to a nitrocellulose membrane (Bio-rad, Hercules, CA, USA), blocked with 5% nonfat milk for 1 h, incubated with primary antibodies against MOAP-1 (1:1000, Sigma, Saint Louis, MO, USA), BDNF (1:1000, Abcam, Cambridge, UK), BAX (1:100, Abcam, Cambridge, UK), and β-actin (1:1000, Cell Signaling Technologies Danvers, MA, USA) at 4 °C overnight, and then incubated with horse radish peroxidase (HRP)-conjugated anti-rabbit or mouse IgG (Millipore, Billerica, MA, USA) at room temperature for 1 h. Signals were detected using Luminata Forte or Crescendo Western HRP substrate (Millipore, Billerica, MA, USA).

### Immunofluorescence Histochemistry

Mice were anesthetized with ketamine/medetomidine and perfused thoroughly with 0.1 M PBS through the heart. Brains were removed and post-fixed with 4% paraformaldehyde (Sigma, Saint Louis, MO, USA) solution overnight at 4 °C, and then dehydrated in 10% sucrose for 1 day followed by 20% sucrose until use. Brain tissues were frozen on dry ice for 15 min and then embedded in OCT compound (Sakura Finetek, Torrance, CA, USA). Coronal sections (20 μm) were cut in cryostat (Leica Biosystems, Buffalo Grove, IL, USA), incubated in 5% goat serum for 1 h to block non-specific binding and then incubated in primary antibodies at 4 °C overnight. Primary antibodies used were antibodies against TPH2 (1: 200, Novus Biological, Littleton, CO, USA), BDNF (1:100, Abcam, Cambridge, UK), BAX (1:10, Abcam, Cambridge, UK), tropomyosin-related kinase B (TrkB) (1:100, R&D Systems, Minneapolis, MN, USA), 5-HT_2A_ receptor (1:100, Abcam, Cambridge, UK), NeuN (1:20, Millipore, Billerica, MA, USA), glial fibrillary acidic protein (GFAP) (1:200, Millipore, Billerica, MA), and Iba-1 (1:100, Wako Chemicals, Tokyo, Japan). Sections were washed in 0.1 M PBS and incubated with Alexa Fluor 488 or 555-conjugated secondary antibodies (1:200, Life Technologies, Carlsbad, CA, USA) for 1 h at room temperature and followed by incubation with 4′,6-diamidino-2-phenylindole dihydrochloride (DAPI, Sigma-Aldrich,, Saint Louis, MO, USA, 0.5 μg/ml in PBS) for 5 min. Sections were then mounted with ProLong gold antifade reagent (Life Technologies, Carlsbad, CA, USA) and fluorescent images captured under a confocal microscope (Olympus, Tokyo, Japan).

### Bromodeoxyuridine (BrdU) Labelling

After 3d-RFSS, mice were intraperitoneally injected with BrdU (150 mg/, Sigma) solution. Animals were anesthetized with ketamine/medetomidine 24 h later and perfused thoroughly with 0.1 M PBS through the heart. The brain tissues were collected and post-fixed with 4% PFA solution overnight at 4 °C, and then dehydrated in 10% sucrose for 1 day followed by 20% sucrose until use. Coronal sections (20 μm) were cut in the cryostat chamber through the dentate gyrus (− 1.34 to − 3.80 mm from Bregma, *n* = 4). Every 12th section (10 sections from each mouse brain) was used for BrdU staining. Briefly, sections were incubated with 1 N HCl for 30 min at 45 °C, washed with 0.1 M PBS, and blocked with 5% goat serum for 1 h. BrdU antibody (1:100, Millipore) was applied overnight at 4 °C. Then the sections were washed with PBS and incubated with the Alexa Fluor 555-conjugated secondary antibodies (1:200) for 1 h at room temperature. Images were acquired under a confocal microscope. Other sections were used for Doublecortin (DCX, 1:200, Abcam) and Ki67 (1:200, Abcam) immunostaining as described above.

### Counting of Immunopositive Cells

Cell quantification was performed in a blinded manner by experimenter who could only identify samples by a code number. For TPH2 and BDNF immunostaining, five sections of the DRN (− 4.6 to − 4.9 mm from Bregma) per mouse were analyzed to obtain a mean count. Per field of view was quantified for all figures of TPH2 (*n* = 3–6). BDNF quantification in Fig. 5a was performed per field of view in Fig. 5a (top panel, *n* = 4–5). For BrdU, Ki67, and DCX immunostaining, all immunostained cells in the subgranular zone and the granule cell layer were counted. The total number of BrdU, Ki67, and DCX-labeled cells per dentate gyrus was obtained by multiplying the total number of counted positive cells by 12. “*n*” refers to the number of mice in each experimental group and not brain sections.

### Plasma Corticosterone Assay

Blood (*n* = 4–5) was collected from mice as described earlier and the plasma was diluted 1:40. Corticosterone level was measured using an enzyme immunoassay (ELISA) kit (Enzo Life Sciences, Farmingdale, NY, USA) according to the manufacturer’s instructions.

### Brain 5-HT Levels

5-HT levels in the midbrain (*n* = 4) were measured by ELISA (Enzo Life Science, Farmingdale, NY, USA). Tissues were homogenized in PBS (100 mg/ml) and stored at − 20 °C overnight. After two freeze-thaw cycles, homogenates were centrifuged and the supernatants were collected for ELISA assay according to manufacturer’s instruction.

### 5-HT Receptor Expression

Total RNA was extracted from the midbrain (*n* = 3–4) using TRIzol Reagent (Invitrogen, Carlsbad, CA, USA) and reverse-transcribed into cDNA using RevertAID First Strand cDNA Synthesis Kit (Thermo Fisher Scientific, Rockford, USA). Quantitative real-time PCR was performed using a Step One Plus Real-Time PCR System (Applied Biosystem, CA, USA) and FastStart Universal SYBR Green Master (Rox) (Roche Diagnostics, IN, USA). Relative mRNA expression was estimated by normalization with GADPH expression. The following primers were used: For 5-HT_1A_ receptor, forward 5′-GGAGCGGGCACCAGCTTCGGAACA-3′ and reverse 5′-CACTGTCTTCCTCTCACGGGCCAA-3′; for 5-HT_1B_ receptor, forward 5′-AAGAAACTCATGGCCGCTAGGGAG-3′ and reverse 5′-GCGTATCAGTTTGTGGAACGCTTG-3′; for 5-HT_2A_ receptor, forward 5′-GGGTACCTCCCACCGACAT-3′ and reverse 5′-AGGCCACCGGTACCCATAC-3′, for GADPH, forward 5′-TCAACGGGAAGCCCATCA-3′ and reverse 5′-CTCGTGGTTCACACCCATCA-3′. Gene copy numbers were expressed with the comparative CT method for relative gene expression.

### Statistical Analyses

**Fig. 1:**
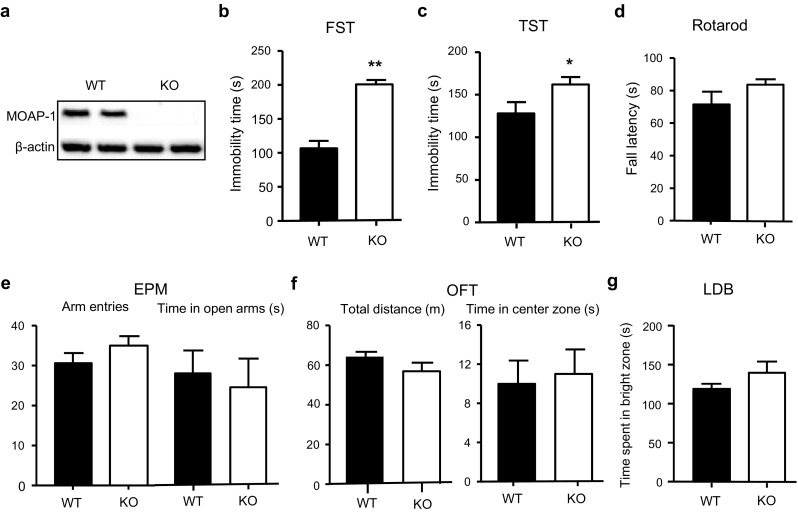
Performance of modulator of apoptosis 1 (MOAP-1)^−/−^ (KO) and wild-type control (WT) mice (age 3–6 months) in various behavioral tests. **a** Western blot showing a lack of MOAP-1 protein expression in the cerebral cortex of MOAP-1^−/−^ mice. **b** Immobility time in the forced swimming test (FST), *n* = 15–17. **c** Immobility time in the tail suspension test (TST), *n* = 15–18. **d** Fall latency in the rotarod test, *n* = 14. **e** Total number of arm entries and time spent in open arms on the elevated plus maze (EPM), *n* = 10. **f** Total distance traveled and time spent in the center zone in the open field test (OFT), *n* = 10–12. **g** Time spent in bright zone in the light dark box (LDB), *n* = 15. Data are presented as mean ± SEM. Total trial time was 4 min for FST, 10 min for OFT, and 5 min for all others. *P < 0.05, **P < 0.01 against WT by independent two-tailed *t* test

All data analyses were conducted using GraphPad Prism 5 software. Data were checked for normality using D′Agostino-Pearson omnibus test before statistical analysis. Normally distributed data were analyzed using independent sample two-tailed *t* test to compare two groups, one-way analysis of variance (ANOVA) to compare multiple groups, followed by post-hoc analysis with Bonferroni correction or two-way ANOVA with repeated measures followed by post hoc analysis with Bonferroni correction. Mann–Whitney *U* test was used for nonparametric data set which failed the normality test (only time in center zone in open field test (OFT), Fig. 1f). Statistical significance was defined as *P* < 0.05.

## Results

### Depression-Like Behavior in Young MOAP-1^−/−^ Mice

Deletion of MOAP-1 gene in MOAP-1^−/−^ mice was verified by Western blotting as no MOAP-1 protein expression was detected in the cerebral cortex (Fig. 1a). In FST, the immobility times were significantly raised in young MOAP-1^−/−^ mice when compared to age-matched WT control mice (Fig. 1b). Similar results were obtained in TST (Fig. 1c). On the contrary, no significant differences were observed between MOAP-1^−/−^ and WT controls in the rotarod test (Fig. 1d), EPM (Fig. 1e), OFT (Fig. 1f), and light dark box (LDB) (Fig. 1g). Thus, MOAP-1^−/−^ mice appear to exhibit depression-like behavior while their anxiety levels were unaltered. As BAX^−/−^ mice are also known to exhibit increased immobilization time in FST [4], we checked the expression of BAX in MOAP-1^−/−^ mice and found normal BAX expression when compared to WT mice (Supplementary Fig. S1A).

**Fig. 2:**
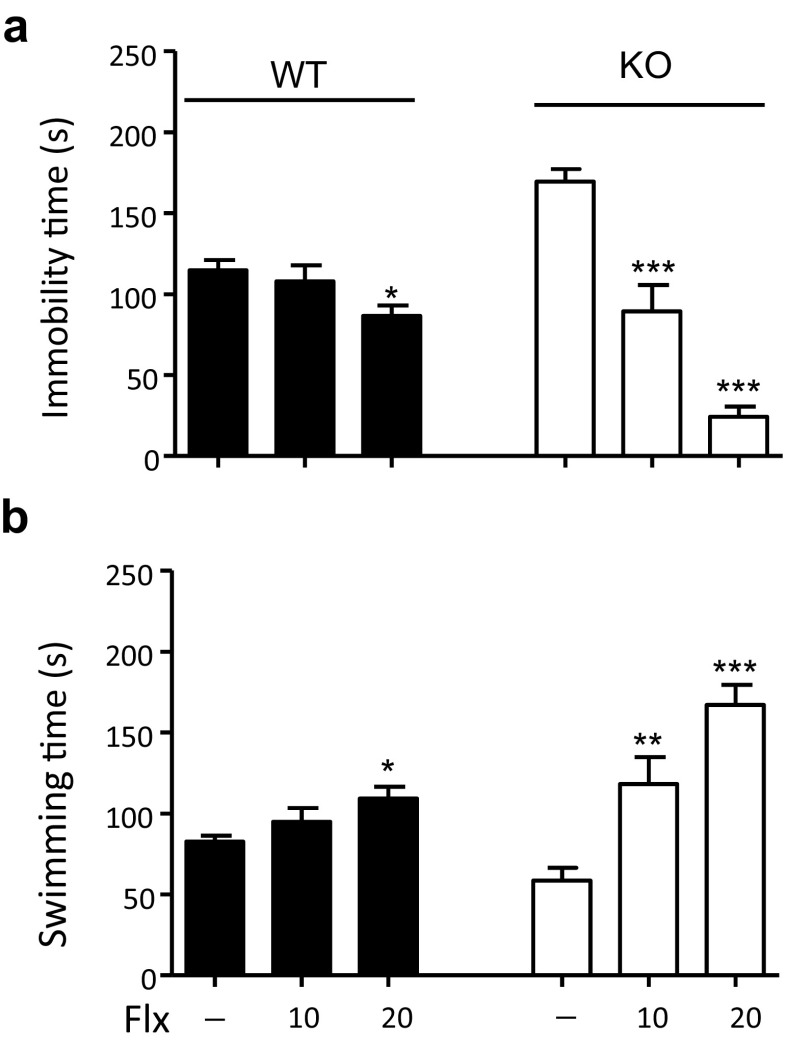
Effects of fluoxetine (Flx) on the FST in modulator of apoptosis 1 (MOAP-1)^−/−^ (KO) and wild-type control (WT) mice. Mice (*n* = 8–10, 3–6 months old) were administered Flx (10 or 20 mg/kg) 30 min before the forced swimming test. Duration (s) of the immobility (**a**) and swimming (**b**) are presented as mean ± SEM. Statistical analysis was performed by one-way ANOVA: **a** For WT mice, *F* = 3.624, *P* < 0.05. For MOAP-1^−/−^ mice, *F* = 58.61, *P* < 0.001. **b** For WT mice, *F* = 3.531, *P* < 0.05. For MOAP-1^−/−^ mice, *F* = 20.90, *P* < 0.001. *P < 0.05, **P < 0.01, and ***P < 0.001 against untreated control by post hoc analysis with Bonferroni correction

When mice were treated with a single dose of Flx (10 or 20 mg/kg i.p.) 30 min before FST, it was observed that, at the lower dose, Flx did not alter the immobilization time of WT mice when compared to the saline-treated group, but markedly reduced the high immobilization time of MOAP-1^−/−^ mice. At the higher dose, Flx significantly decreased immobilization time in both groups of mice when compared to the saline-treated as well as the lower dose group (Fig. 2a). The effects of Flx on swimming time was the exact opposite whereby swimming time was significantly increased at the higher dose in the WT mice and markedly increased at both doses in the KO mice (Fig. 2b). The observed effects of Flx in WT mice are consistent with previous findings [35–39] where a single dose of Flx (10–30 mg/kg) administered by intraperitoneal injection 30 min prior to FST reduced immobilization time in C57BL/6 mice. The fact that Flx at 10 mg/kg administered acutely shortly before FST totally abolished the increased immobilization time in MOAP-1^−/−^ mice but had no effects in the WT mice strongly suggests that serotonergic transmissions in the MOAP-1^−/−^ mice may be impaired.

**Fig. 3:**
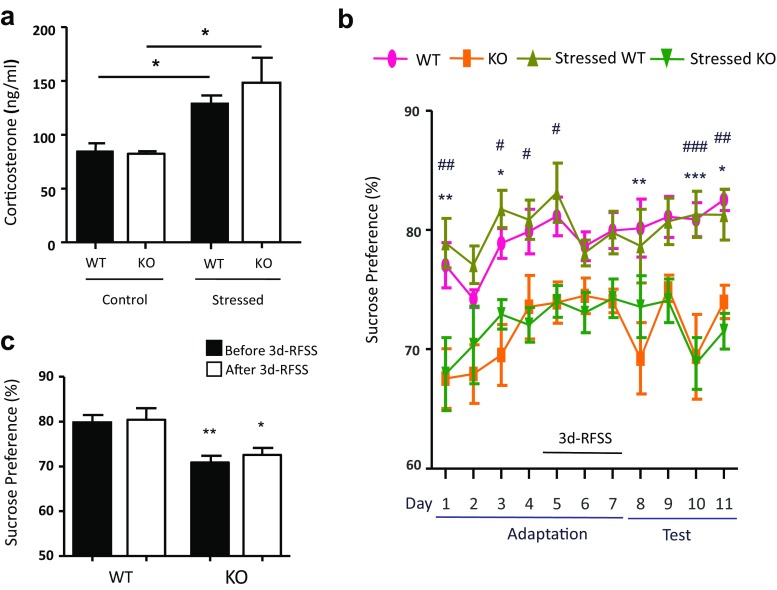
Effects of 3d-RFSS on plasma corticosterone levels and sucrose preference in modulator of apoptosis 1 (MOAP-1)^−/−^ (KO) and wild-type control (WT) mice (age 3–6 months). **a** Plasma corticosterone levels with or without stress treatment, *n* = 4–5. Data are presented as mean ± SEM. Statistical analysis was performed by one-way ANOVA: *F* = 5.848, *P* < 0.05. *P < 0.05 against control WT or MOAP-1 KO by Bonferroni correction. **b** Sucrose preference measured daily in WT and MOAP-1^−/−^ mice over 11 days. 3d-RFSS was applied from days 5–7. Data are presented as mean ± SEM, *n* = 8. Statistical analysis was performed by two-way ANOVA with repeated measures followed by Bonferroni correction: day factor: *F* = 4.54, *P* < 0.001; group factor: *F* = 16.91, *P* < 0.001; day × group interaction: *F* = 0.90, *P* = 0.6274. *P < 0.05, **P < 0.01, and ***P < 0.001 for WT vs KO; #*P* < 0.05, ##*P* < 0.01, and ###*P* < 0.001 for stressed WT vs stressed KO. **c** Comparison between before stress (combined data for days 1–4) and after stress (combined data for days 8–11). Data are presented as mean ± SEM, *n* = 8. Statistical analysis was performed by one-way ANOVA followed by Bonferroni correction: *F* = 8.254, *P* < 0.001. *P < 0.05, **P < 0.01 when compared to the corresponding WT controls

To further confirm the depression-like behavior in these MOAP-1^−/−^ mice, we compared sucrose preference between MOAP-1^+/+^ and MOAP-1^−/−^ mice. As repeated forced swimming stress has been reported to induce anhedonia [40] as well as not to induce anhedonia [41] in the SPT, we therefore also investigated sucrose preference with and without 3d-RFSS. Figure 3a shows that 3d-RFSS, as expected, induced a significant increase in plasma corticosterone levels in both WT control and MOAP-1^−/−^ mice. The SPT results (Fig. 3b) clearly showed a significantly lower sucrose preference, thus indicating anhedonia, in the MOAP-1^−/−^ mice when compared to WT mice during both the adaptation and test periods. Figure 3c presents the combined data for the 4 days before stress and the 4 days after stress confirming that MOAP-1^−/−^ mice had a significantly lower sucrose preference than the WT mice before and after stress treatment. These results confirmed the depression-like behavior in MOAP-1^−/−^ mice as shown in the FST and TST. However, no differences in sucrose preference were observed before and after 3d-RFSS in both groups of mice.

### Absence of Serotonergic Stress Response in the DRN of MOAP-1^−/−^ Mice

**Fig. 4:**
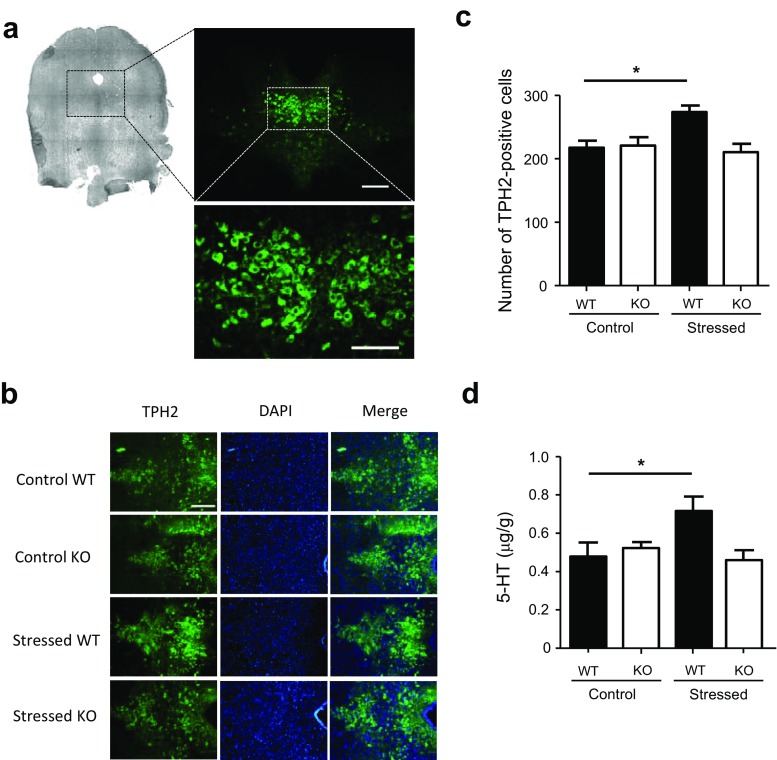
Effects of stress on the expression of tryptophan hydroxylase (TPH) 2 in the dorsal raphe nucleus (DRN) of modulator of apoptosis 1 (MOAP-1)^−/−^ (KO) and wild-type control (WT) mice (age 3–6 months). **a** Brightfield image of a coronal section of mouse midbrain at − 4.6 mm from Bregma (left panel). TPH2 immunofluorescent staining can be seen concentrated in the DRN. Scale bar = 200 μm (middle panel) and 100 μm (right panel). **b** Representative photomicrographs of TPH2 immunofluorescent staining in the DRN of the WT and MOAP-1^−/−^ mice with or without stress treatment. Scale bar = 200 μm. **c** Number of TPH2 immunopositive cells in the DRN in WT and MOAP-1^−/−^ mice with or without stress treatment, *n* = 3–6. Data are presented as mean ± SEM. ANOVA: *F* = 5.917, *P* < 0.05. *P < 0.05 for control vs stressed WT mice by Bonferroni correction. **d** 5-HT levels in midbrain of WT and MOAP-1^−/−^ mice with or without stress treatment, *n* = 4. Data are presented as mean ± SEM. ANOVA: *F* = 4.366, *P* < 0.05. *P < 0.05 for control vs stressed WT mice by Bonferroni correction

Tryptophan hydroxylase 2 (TPH2) is a neuron-specific isoform [42, 43] of TPH that catalyzes the rate-limiting step in the synthesis of 5-HT in the brain. It is thus used as a marker for serotonergic neurons, which are concentrated in the DRN in the midbrain region [44] (Fig. 4a). Repeated forced swimming used as a stressor was observed to increase the mRNA levels of TPH2 in the midbrain [44, 45]. When the expressions of TPH2 in the DRN were compared between MOAP-1^−/−^ and MOAP-1^+/+^ mice, we did not observe any significant difference. However, following 3d-RFSS, a significant increase in TPH2-immunostaining was observed in the DRN of WT mice (Fig. 4b) that could be attributed to an increase in the number of TPH2-immunopositive cells in these WT mice (Fig. 4c). In contrast, the number of TPH2-immunopositive cells remained the same in MOAP-1^−/−^ mice after stress. In support, tissue 5-HT levels were also increased in the midbrain in the MOAP-1^+/+^ mice, but not the MOAP-1^−/−^ mice following 3d-RFSS (Fig. 4d). In addition, we observed no significant changes in the mRNA expressions of 5-HT_1A_, 5-HT_1B,_ and 5-HT_2A_ receptors in these mice with or without 3d-RFSS (Supplementary Fig. S2). Therefore, it may be concluded that MOAP-1 is involved in the stress response and when it is deficient, the MOAP-1^−/−^ mice fail to mount a serotonergic response to stress. It should be noted that similar changes in TPH2 expression in the DRN were not observed following a single episode of forced swimming (SEFS) (Supplementary Fig. S3).

### Age Difference in Serotonergic Stress Response

**Fig. 5:**
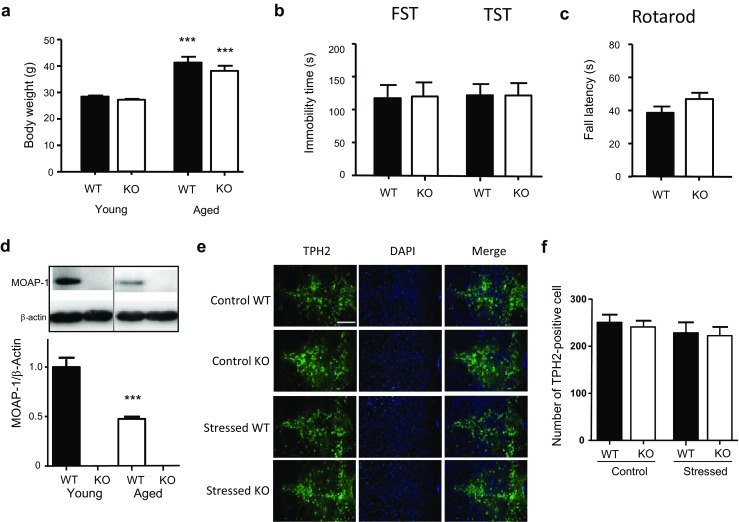
Behavior and stress response in aged (22–26 months old) modulator of apoptosis 1 (MOAP-1)^−/−^ (KO) and wild-type control (WT) mice. **a** Comparison of body weight of young (*n* = 19) and aged (*n* = 12) MOAP-1^+/+^ and MOAP-1^−/−^ mice. One-way ANOVA: *F* = 45.65, *P* < 0.0001. ***P < 0.001 against the corresponding young mice by Bonferroni correction. **b** Performance of aged mice in the FST and TST, *n* = 14–15. No statistical significance observed. **c** Fall latency of aged mice in the rotarod test, *n* = 15–17. No statistical significance observed. **d** Western blot showing decreased MOAP-1 protein expression in the midbrain region of aged WT in comparison to young (3–6 months) WT mice. ***P < 0.001 against young WT mice by independent two-tailed *t* test. **e** Representative photomicrographs of tryptophan hydroxylase (TPH) 2 immunofluorescent staining in the dorsal raphe nucleus (DRN) of the aged WT and MOAP-1^−/−^ mice with or without 3d-RFSS treatment. Scale bar = 200 μm. **f** Number of TPH2 immunopositive cells in the DRN in WT and MOAP-1^−/−^ mice, *n* = 3–4. ANOVA: *F* = 0.515, not significant. Data are presented as mean ± SEM

To investigate the effects of age, we studied aged mice at 22–26 months of age. Figure 5a shows that there was no significant difference in body weight between the aged WT and MOAP-1^−/−^ mice. On FST and TST, the immobility times in aged mice were the same between WT and MOAP-1^−/−^ mice at a level more comparable to that for young WT mice rather than young MOAP-1^−/−^ mice (Fig. 5b). As expected, these aged mice exhibited inferior performance in the rotarod test when compared to young mice, indicating diminished motor function and/or coordination, but no significant difference was observed between aged WT and aged MOAP-1^−/−^ mice (Fig. 5c). A check on the MOAP-1 expression revealed that the protein level of MOAP-1 diminished by about 50% in aged WT mice when compared to young mice (Fig. 5d). In contrast, no significant alteration in BAX expression was observed in both groups over age (Supplementary Fig. S1B). Interestingly, stressed aged WT mice did not exhibit any increase in the number of TPH2 immunopositive cells in the DRN (Fig. 5e/f) in contrast to young WT mice (Fig. 4b/c). In other words, like MOAP-1^−/−^ mice, aged WT mice failed to exhibit the serotonergic stress response in the DRN.

### Serotonergic Stress Response Is Associated with Decreased Expression of BDNF

**Fig. 6:**
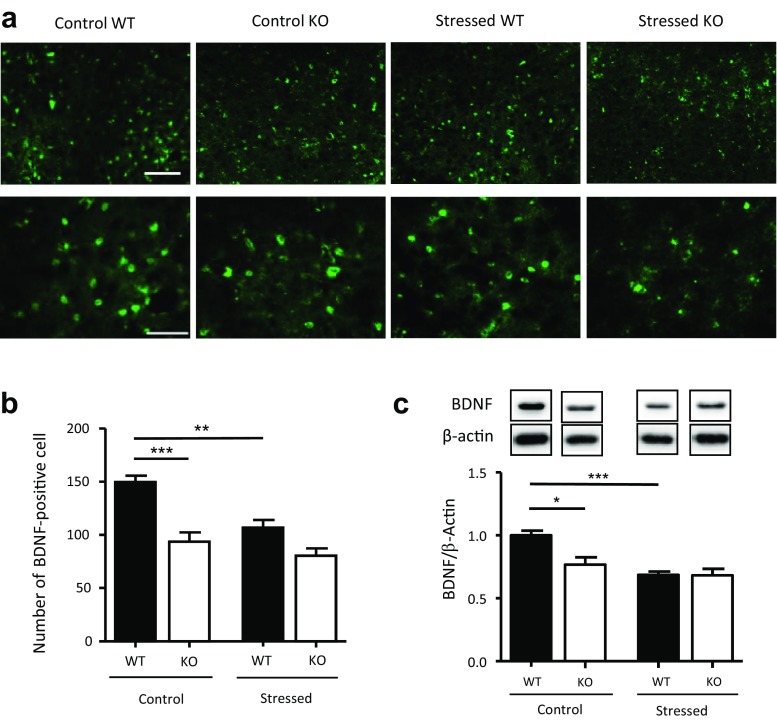
Effects of stress on the expression of brain-derived neurotrophic factor (BDNF) in the dorsal raphe nucleus (DRN) of modulator of apoptosis 1 (MOAP-1)^−/−^ (KO) and wild-type control (WT) mice (age 3–6 months). **a** Representative photomicrographs of BDNF immunofluorescent staining in the DRN of the WT and MOAP-1^−/−^ mice with or without stress treatment. Scale bar = 100 μm (top panel) or 50 μm (bottom panel). **b** Number of BDNF immunopositive cells in the DRN in WT and MOAP-1^−/−^ mice with or without stress treatment, *n* = 4–5. Data are presented as mean ± SEM. ANOVA: *F* = 17.38, *P* < 0.001. **P < 0.01 and ***P < 0.001 against control WT by Bonferroni correction. **c** Expression of BDNF in the midbrain region by Western blot analysis, *n* = 3–4. Data are presented as mean ± SEM. ANOVA: *F* = 10.52, *P* < 0.05. *P < 0.05, ***P < 0.001 by Bonferroni correction. Representative blot bands of the corresponding groups are shown in the top panel

The number of BDNF-immunopositive cells in the DRN of MOAP-1^−/−^ mice was observed to be reduced when compared to that in WT mice (Fig. 6a/b). When subjected to 3d-RFSS, WT mice responded with a downregulation of BDNF expression. In contrast, BDNF expression in MOAP-1^−/−^ mice remained unchanged following stress (Fig. 6b). BDNF expression in the midbrain region including the DRN obtained by Western blotting provided corroborating data (Fig. 6c).

**Fig. 7:**
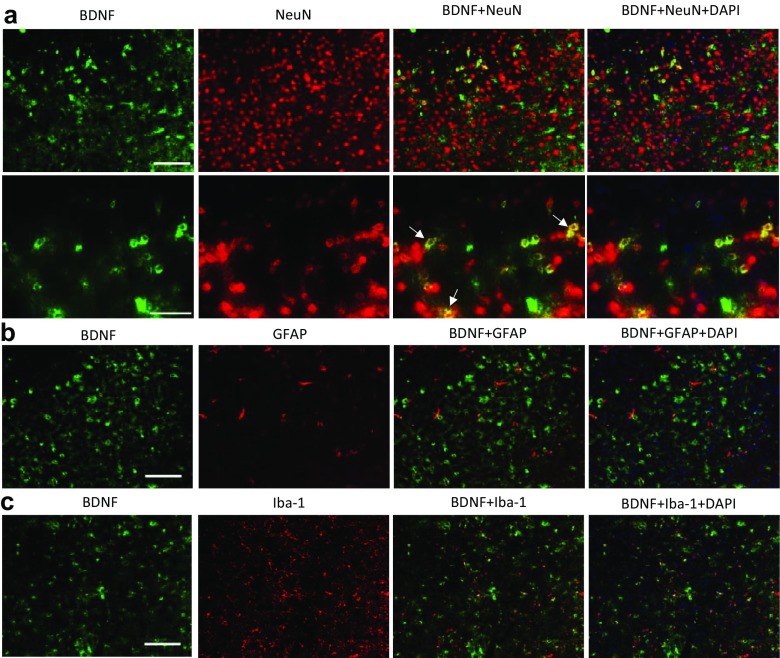
Cellular localization of brain-derived neurotrophic factor (BDNF) in the dorsal raphe nucleus (DRN) of wildtype mice. **a** Double staining of BDNF with NeuN. Scale bar = 100 μm (top) and 50 μm (bottom). White arrows indicate colocalization. **b** Double staining of BDNF with glial fibrillary acidic protein (GFAP). Scale as in (**a**), top panel. **c** Double staining of BDNF with induction of brown adipocytes 1 (Iba-1). Scale as in (**a**), top panel

**Fig. 8:**
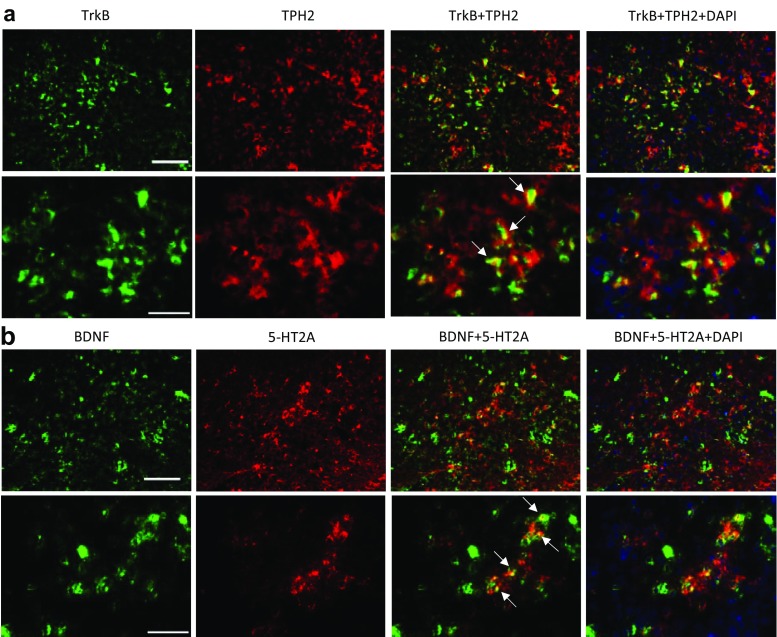
Double immunostaining of **a** tropomyosin-related kinase B (TrkB) and tryptophan hydroxylase 2 (TPH2) and **b** brain-derived neurotrophic factor (BDNF) and 5-hydroxytryptamine (5HT)_2A_ receptor in the dorsal raphe nucleus (DRN) of wild-type mice. Scale bars = 100 μm (top) or 50 μm (bottom). White arrows indicate colocalization

The BDNF-immunopositive cells showed colocalization with NeuN (Fig. 7a), but not with GFAP (Fig. 7b) and Iba-1 (Fig. 7c), indicating that BDNF is expressed in neurons. In addition, BDNF immunoreactivity does not colocalize with either TPH2 or tropomyosin-related kinase B receptor (TrkB) in the DRN (Supplementary Fig. S4). On the contrary, there was a significant extent of colocalization of TPH2 and TrkB immunoreactivity in the DRN (Fig. 8a). The neuronal nature of TrkB-immunopositive cells were further supported by colocalization of TrkB with NeuN but not GFAP and Iba-1 staining (Supplementary Fig. S5). Furthermore, some colocalization of BDNF and 5HT_2A_ receptors was also observed (Fig. 8b). Similar observations were obtained with MOAP-1^−/−^ mice as described for WT mice. These results indicate that crosstalk between BDNF and 5-HT is highly probable in the DRN.

### Hippocampal Neurogenesis

**Fig. 9:**
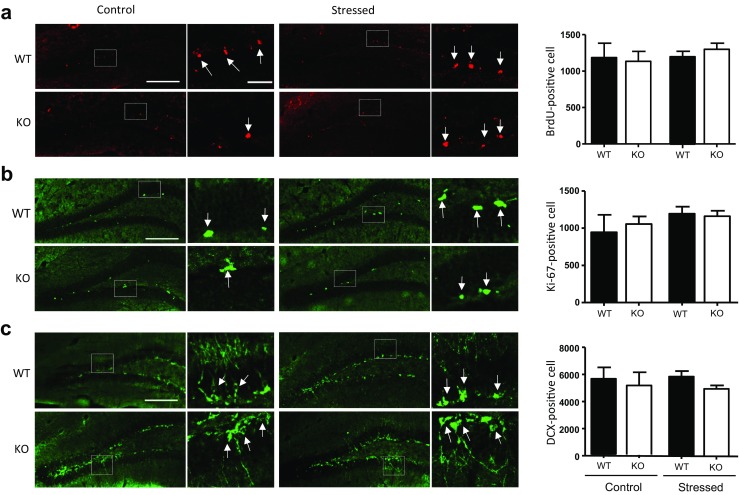
Neurogenesis in the hippocampus of modulator of apoptosis 1 (MOAP-1)^−/−^ (KO) and wild-type control (WT) mice following 3d-RFSS (age 3–6 months). Immunohistochemical staining of **a** BrdU, **b** Ki67, and **c** DCX in the dentate gyrus. The right panels are representative photomicrographs. Arrows indicate positively stained cells. The white box indicates the area where the high magnification photomicrograph was taken. Scale bar = 200 or 40 μm. The left panels present the number of positively stained cells. Data are presented as mean ± SEM, *n* = 4. Statistical analysis performed by one-way ANOVA: **a** *F* = 0.2749, **b** *F* = 0.6245, **c** *F* = 0.3487, no statistical significance

To investigate hippocampal neurogenesis, we performed immunostaining for BrdU, Ki67 (a marker for cell proliferation), and doublecortin (DCX, a neuronal precursor cell marker) in the dentate gyrus (DG) in WT and MOAP-1^−/−^ mice with and without stress. Figure 9 shows that no significant changes in the number of BrdU-positive, Ki67-positive, and DCX-positive cells were observed in all groups, indicating that WT and MOAP-1^−/−^ mice have the same level of neurogenesis in the DG and that 3d-RFSS does not influence neurogenesis at a detectable level under the experimental conditions used.

## Discussion

In this study, we have demonstrated that MOAP-1^−/−^ mice exhibit depression-like behavior in FST and TST, as well as SPT. 3d-RFSS upregulated TPH2 expression in the DRN in an age- and MOAP-1-dependent manner. BDNF was downregulated concomitantly to the TPH2 upregulation. The present results suggest functional interplay of BDNF and 5-HT in the DRN. However, 3d-RFSS did not significantly alter hippocampal neurogenesis.

Stress has been shown to be a risk factor of depression [46]. Both FST and TST are acute stress models of depression that are widely used to deduce depression-like behavior and to test drugs for antidepressant-like effects [47]. We presently observed that MOAP-1^−/−^ mice exhibit depression-like behavior in both tests (Fig. 1b/c) and that such behavior can be reversed by Flx (Fig. 2). SSRIs typically reduce immobility and increase swimming in the FST with minimal effects on climbing or thrashing [48]. As Flx was administered acutely, the reversal of the increased immobility in MOAP-1^−/−^ mice is most likely a result of 5-HT reuptake inhibition, indicating impaired serotonergic transmission in these mice. Krahe et al. [12] reported that BAX^−/−^ mice exhibited increased FST immobility time and these authors suggested that abnormal emotional states may result from disruption of programmed cell death. Our present observations with MOAP-1^−/−^ mice provide further credence to this idea.

While both BAX^−/−^ and MOAP-1^−/−^ mice showed depression-like behavior, they differ in another aspect. Luedke et al. [11] reported that BAX^−/−^ mice exhibited reduced anxiety on the EPM when compared to WT controls, while MOAP-1^−/−^ mice did not appear to have an altered anxiety state (Fig. 1e–g), suggesting that MOAP-1 and BAX deficiencies may not lead to identical effects on programmed cell death and thus differing effects on functional parameters. Additional studies would be necessary to more fully understand the role of MOAP-1 in depression. However, based on the high level of MOAP-1 expression, Takaji et al. [8] suggested that such abundance is incompatible with the notion that MOAP-1 acts only as an apoptotic modulator in the brain. This is supported by in vitro observations that over-expression of MOAP-1 does not induce apoptosis in SY5Y human neuroblastoma cells [1] and Neuro2a mouse neuroblastoma cells [8]. Thus, it is also a possibility that MOAP-1 participates in regulating brain functions apart from its role in apoptosis. The present observations provide the first piece of evidence that links MOAP-1 to brain function.

Both depression and anxiety disorders involve dysfunction of the stress response [49] and the serotonergic systems have been a major therapeutic target for the treatment of stress disorders [21]. Accumulating evidence indicates that the highly adaptable and inducible neuron-specific TPH2 plays an active role in stress response and stress disorders [17, 44, 50–58]. As it has been reported that stress response could occur rapidly within 30 min in the brain [59] following acute stress, we examined TPH2 expression in the DRN after a single episode of forced swimming but failed to observe any changes (Supplementary Fig. S3). However, when mice were exposed to 3d-RFSS, a significant increase in TPH2-immunopositive cells was observed in WT control mice (Fig. 4c), which is supported by the increase in tissue 5-HT levels in the midbrain (Fig. 4d) but there were no observed changes in the mRNA expressions of 5-HT_1A_, 5-HT_1B_, and 5-HT_2A_ receptors (Supplementary Fig. S2). Effects of forced swimming stress on 5-HT levels reported in the current literature vary considerably. Shishkina et al. [45] reported no change in the midbrain and cortex, but a decrease in the hippocampus after 2d-RFSS, while Kirby and Lucki [60] reported an increase in the striatum and a decrease in the lateral septum after the first day of 2d-RFSS and no change from baseline in both regions on the second day. Abbas et al. [61] reported an increase in the hippocampus after SEFS but no change after 7d-RFSS, while Briones-Aranda et al. [62] reported an increase in the hippocampus, a decrease in thalamus-hypothalamus, and no change in the brain stem after SEFS. On the other hand, 5-HT_1A_ expression has been reported to be unchanged in the midbrain after 2d-RFSS [45]. In contrast, it had also been reported to be decreased in the DRN and hippocampus but increased in the thalamus/hypothalamus and amygdala after SEFS [62]. It was also reported that 2-week restraint stress decreased 5-HT_1A_ expression in the hippocampus [63], but acute immobilization stress did not alter 5-HT_2A_ receptor binding in the cortex and hippocampus [64]. Overall, these observations show that changes in the serotonergic systems in response to forced swimming and other forms of stress vary from region to region and are highly dependent on the duration/severity of the stress regime.

The present observation of an increase in TPH2-immunopositive cells is, however, consistent with previously reported increase in TPH2 mRNA levels in the midbrain region following two exposures to forced swimming [44, 59]. Many factors including environmental factors, hormones, growth factors, and drugs have been repeatedly demonstrated to affect TPH2 gene expression [55]. Up-regulation of TPH2 induced by chronic Flx treatment appeared to correlate to its antidepressant effects [53], thus TPH2 up-regulation may be a counter depression measure. Consistently, increases in the number of serotonergic neurons and TPH2 expression have been observed in postmortem DRN of depressed suicides [65, 66]. It has been suggested that the elevated TPH2 expression may reflect a homeostatic response to 5-HT deficiency [65]. However, an earlier report indicated that the suicide group might have significantly fewer DRN neurons expressing SERT, but a higher expression of SERT per serotonergic neuron indicating hypofunction of the serotonergic system in the DRN of depressed suicides [67].

On the contrary, no increase in TPH2-immunopositive cells and tissue 5-HT levels occurred in MOAP-1^−/−^ mice (Figs. 4 and 5). This may be interpreted as an inability of MOAP-1^−/−^ mice to mount such a serotonergic compensatory response in the DRN under stress. As the depression-like behavior (increased immobility time) exhibited by MOAP-1^−/−^ mice may be a result of impaired serotonergic transmission based on its reversal by acutely administered Flx, this may suggest that such a stress response in the DRN is contingent to having unimpaired serotonergic functions. Thus MOAP-1 deficiency may be a possible cause of serotonergic impairment.

In aged mice, contrary to young mice, no differences were observed between WT and MOAP-1^−/−^ mice in FST and TST (Fig. 5b). This may be explained, at least in part, by the markedly diminished expression of MOAP-1 protein in aged WT mice when compared to young mice (Fig. 5d). Diminished brain function is part of the aging process as evidenced by the observed deficit in rotarod performance (Fig. 5c). Thus, it may be reasonable to expect a significant degree of impairment of serotonergic functions in the aged brain. If so, this may explain the observed lack of stress response in the form of increased TPH2 expression (Fig. 5e/f) in aged WT mice, consistent with the above argument that the stress response requires normal serotonergic transmission. In addition, one might also expect the immobility times to be the same between aged WT and MOAP-1^−/−^ mice, as observed (Fig. 5b), but at a level close to that of young MOAP-1^−/−^ mice. The actual observed level was nearer to the level of young WT mice instead. This may not be readily explained. The present data do not indicate any age-dependent decrease in the FST immobilization time in WT mice but a significant decrease in MOAP-1^−/−^ mice (Figs. 1 and 5). Previous findings also indicated no change in mice of various age groups ranging from 10 [68], 18 [69], to 22–24 [70] months. In contrast, Shoji et al. [71] recently reported that age-related decrease in FST immobilization time in C57BL/6J mice up to 12 months of age. If that is correct, then it is logical to assume further decrease may occur beyond 12 months.

The SPT differs from the FST and TST as it is not a stress-based model but a reward-based model. It is natural for mice to have a sucrose preference of around 80–90%. A lower sucrose preference would indicate an impairment or inability to experience pleasure which is a symptom of depression. The present results show that MOAP-1^−/−^ mice have a significantly reduced sucrose preference of around 70% when compared to the WT mice (Fig. 3b/c), corroborating with the FST and TST results (Fig. 1b/c). However, the sucrose preference was not altered by 3d-RFSS in both groups of mice. Serchov et al. [40] reported anhedonia immediately after 5d-RFSS which was attenuated by increasing adenosine A_1_ receptor expression in the brain. In contrast, Mul et al. [41] reported that 5d-RFSS did not affect sucrose preference in C57BL/6J mice and enhanced sucrose preference in BALB/cJ mice. These authors suggested that differences in the experimental animals such as genetic background may have caused the discrepancy. Our observations appear to agree with this suggestion as our mice have the same C57/BL6 background. Hodes et al. [72] reported that a 6-day subchronic variable stress regime did not alter sucrose preference in C57BL/6J male mice, but decreased it in female mice. In addition, Strekalova and Steinbusch [73] reported that even a severe 4-week chronic stress regime (including continuous exposure to a rat while in a small cage in week 1, daily 2-h restraint stress in week 2, the same treatment as in week 1 in weeks 3 and 4 with the addition of daily 40 min tail suspension in week 4) produced variable reduction in sucrose preference in C57BL/6 mice. Therefore, it does appear that mice exhibit a large variation in their responses in the sucrose preference test based on genetic background, gender, and the nature of the stress regime.

BDNF belongs to a neurotrophin family of growth factors and acts to support cell survival, growth, differentiation, and synaptogenesis [74–77]. BDNF exerts most effects through its specific receptor TrkB, a tyrosine kinase. Binding of BDNF to TrkB causes it to autophosphorylate and interact with signaling effectors via pTyr-binding (PTB)/Src homology 2 (SH2) domains. TrkB activation eventually leads to activation of extracellular signal-regulated kinases (ERK) and phosphoinositide 3-kinase (PI3K) [78, 79]. Through this pathway, BDNF regulates proteins involved in cell survival via both anti-apoptotic (e.g., Bcl-2) and proapoptotic (e.g., BAX) members of the Bcl-2 family of proteins [79]. In early geniculate neurons, it has been reported that BNDF regulates cell survival by inhibiting cell death as BAX deficiency completely eliminated cell loss caused by BDNF deficiency [76].

There is strong evidence that BDNF influences the serotonergic systems [80]. BDNF has been reported to decrease basal level of extracellular 5-HT and K^+^-evoked 5-HT release via activation of TrkB receptors in the hippocampus [28]. Brain-selective depletion of BDNF through genetic mutation can cause significant alterations of 5-HT_2_ receptor-mediated regulation of both excitatory glutamatergic and inhibitory GABAergic transmissions in the basolateral amygdala [29]. The present results demonstrated that under stress, the increase in TPH2 expression in the DRN is associated with a decrease in BDNF in WT mice. This is consistent with observations that TPH2^−/−^ mice have significantly elevated levels of BDNF in the hippocampus and prefrontal cortex [81]. In addition, several reports have documented significant reduction of BDNF gene expression after acute stress in the hippocampus [82, 83] and frontal cortex [84]. However, it has also been reported that acute stress induced an increase in hippocampal BDNF mRNA and protein [85]. On the other hand, MOAP-1^−/−^ mice showed a significantly reduced BDNF expression in the DRN, which remained unchanged when stressed (3d-RFSS), while TPH2 expression was normal and also remained unchanged when stressed (Fig. 6). Moreover, many TPH2-expressing neurons are TrkB-immunopositive (Fig. 8) and TPH2 and BDNF immunoreactivities do not colocalize (Supplementary Fig. S4). These observations, therefore, support the idea that BDNF from non-serotonergic neurons influences serotonergic neurons via TrkB receptors in the DRN, suggesting that the serotonergic stress response may require a BDNF input. Thus, it may be speculated that the depression-like behavior of the MOAP-1^−/−^ mice may be related to the reduced BDNF which in turn blunted the serotonergic stress response in the DRN. Consistently, chronic antidepressant drug treatments regulate the expressions of BDNF and TrkB, and increase the expression of cAMP response element binding protein (CREB) in the hippocampus [26, 86]. Vaidya et al. [87] reported that pretreatment with a selective 5HT_2A_ antagonist significantly blocked the stress-induced decrease in the levels of BDNF mRNA in the hippocampus, but not 5HT_2C_ and 5HT_1A_ antagonists. Adachi et al. [88] recently observed that subchronic desipramine treatment became ineffective in reducing FST immobility time following knockdown of TrkB in the DRN, but remained effective following knockdown of BDNF. Chronic Flx also became ineffective in TrkB knockdown mice. This may indicate that the antidepressant efficacy of SSRIs is dependent on BDNF action on TrkB in the DRN and that BDNF is in excess in this region.

It is also interesting to note that when stressed, MOAP-1^+/+^ mice responded with an increase in TPH2 expression but a decrease in BDNF expression. This may be interpreted as a feedback inhibition on the BDNF expressing neurons by 5-HT. We had demonstrated the colocalization of BDNF and 5-HT_2A_ receptor expression in the DRN (Fig. 8b) but other 5-HT receptor subtypes may also be present. Therefore, speculatively, as both 5-HT and BDNF are known to stimulate hippocampal neurogenesis [21], it appears possible that BDNF may act via 5-HT. Overall, the cause-or-consequence relationship between changes in TPH2 and BDNF presently observed in the DRN is an open question. Further investigations are necessary to further understand the mechanisms by which BDNF-5-HT crosstalk in the DRN influences the stress response.

Studies have also demonstrated a role of apoptosis in depression [89]. Reduction in the volume of cortex and hippocampus, increased cell death, and changes of apoptotic markers were detected in postmortem brains of depressed patients and chronic stress models in animals [90–97]. Antidepressant treatments have been reported to lead to alleviation of cell death and enhancement of neurogenesis [98–100]. Similarly, transgenic mice over-expressing anti-apoptotic gene Bcl-2 decreased neuronal cell death, altered anxiety behavior, and impaired learning ability [101, 102]. In human, perturbations in cell death were associated with schizophrenia and depression [103]. Conversely, increasing adult hippocampal neurogenesis was reported to reduce anxiety and depression-related behavior in mice chronically stressed by corticosterone [104]. However, in the present study, we did not observe any significant differences in hippocampal neurogenesis between WT and MOAP-1^−/−^ mice. In addition, 3d-RFSS also did not alter neurogenesis in both groups of mice (Fig. 9).

In conclusion, MOAP-1^−/−^ mice exhibit increased immobility times when compared to WT controls in FST and TST, both are acute stress models of depression, probably caused by impaired serotonergic functions. In aged MOAP-1^−/−^ and WT mice, the immobilization time do not differ. When subjected to 3d-RFSS, young WT mice show a stress response in the DRN in the form of up-regulation of TPH2-immunopositive serotonergic neurons, which is associated with decreased BDNF expression. However, the serotonergic stress response is absent in MOAP-1^−/−^ mice as well as aged WT mice. Crosstalk between BDNF and 5-HT appears to play an important role in stress response in the DRN. We believe that the present data provide evidence for the first time linking MOAP-1 to brain function. Further investigations are needed to understand the mechanisms involved. Lastly, it should be mentioned that given the heterogeneity of the DRN, it is possible that the effects of stress and MOAP-1 deficiency on TPH2 and BDNF expressions may vary among the DRN subnuclei.

## Electronic supplementary material

ESM 1 (PDF 497 kb)

## Abbreviations

3d-RFSS: 3-Day repeated forced swimming stress
5-HT: 5-Hydroxytryptamine
BAX: Bcl-2-associated X protein
BDNF: Brain-derived neurotrophic factor
DCX: Doublecortin
DG: Dentate gyrus
DRN: Dorsal raphe nucleus
EPM: Elevated plus maze
Flx: Fluoxetine
FST: Forced swimming test
GFAP: Glial fibrillary acidic protein
HPA: Hypothalamus-pituitary-adrenal axis
LDB: Light dark box
MOAP-1: Modulator of apoptosis 1
OFT: Open field test
RASSF1A: Ras association domain family 1A
SEFS: Single episode of forced swimming
SPT: Sucrose preference test
SSRI: Selective serotonin reuptake inhibitor
TNF: Tumor necrosis factor
TPH2: Tryptophan hydroxylase 2
TrkB: Tropomyosin-related kinase B
TST: Tail suspension test
WT: Wild-type
