# Supplementary material for: Absence of Stress Response in Dorsal Raphe Nucleus in Modulator of Apoptosis 1-Deficient Mice
Source: Mol Neurobiol. 2018 Jul 12;56(3):2185–201. doi: 10.1007/s12035-018-1205-7 (PMC6394635; doi:10.1007/s12035-018-1205-7)
Supplement: Supplementary file 1 — (PDF 497 kb) [file 12035_2018_1205_MOESM1_ESM.pdf]

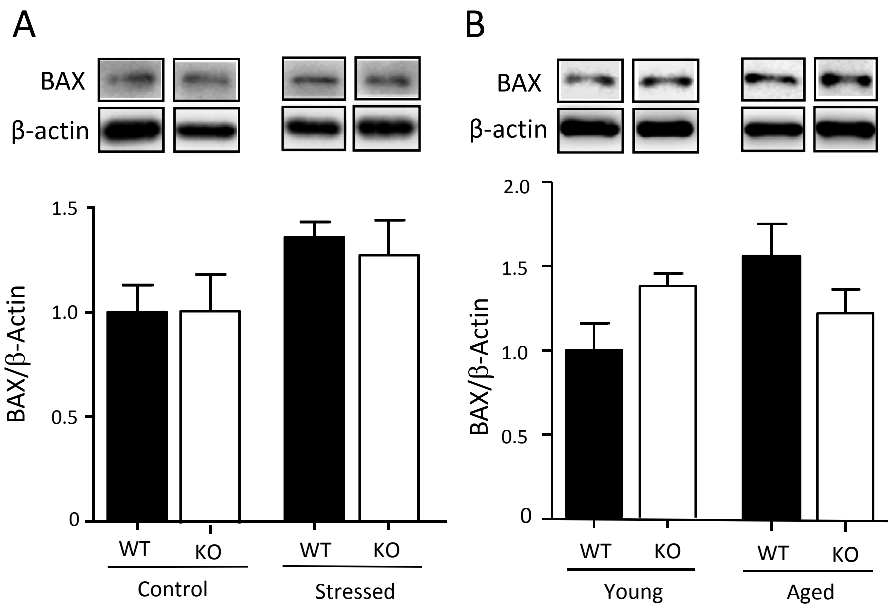

Fig. S1. Expression of apoptosis regulator Bax in the midbrain region of MOAP-1$^{-/-}$ (KO) and wildtype control (WT) mice. Data are presented as Mean±SEM, n = 3-4. (A) No significant changes after repeated forced swimming stress in both WT and MOAP-1$^{-/-}$ mice, ANOVA: F=1.615. (B) No significant changes were observed between young (3-6 months) and aged (22-26 months) mice, ANOVA: F=2.594.

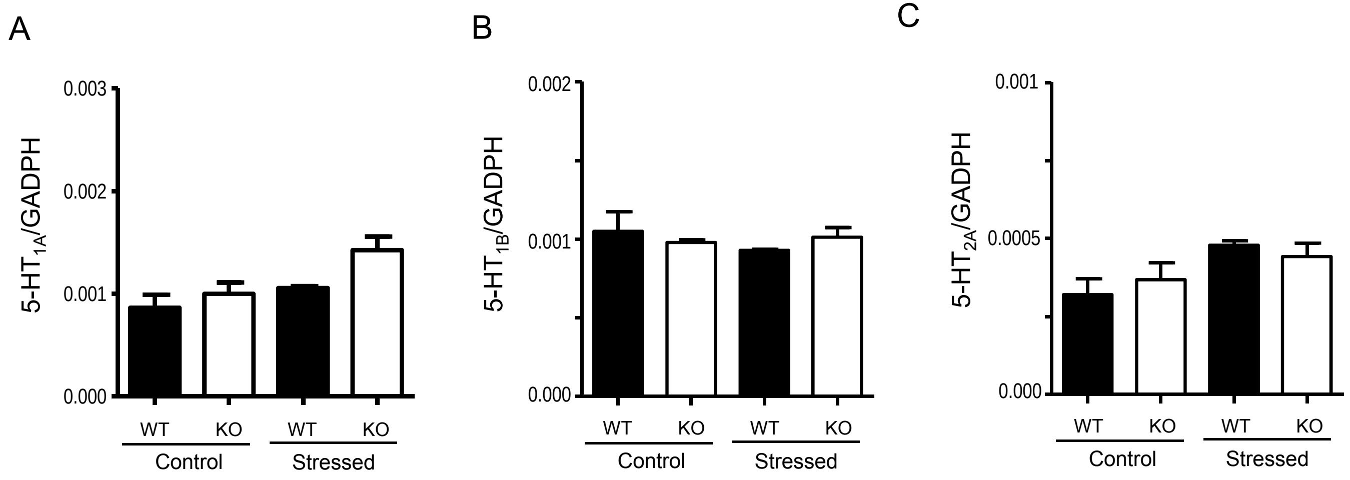

Fig. S2. Relative mRNA expression of (A) 5-HT_{1A}, (B) 5-HT_{1B} and (C) 5-HT_{2A} in the midbrain of WT and MOAP-$1^{-/-}$ (KO) mice with or without 3d-RFSS treatment (n=3-4). Data are presented as Mean±SEM. No significant differences between either WT vs KO or Control vs Stressed. ANOVA, F= 4.910, 0.5047 and 2.774, respectively. Note: Although the F value for A is significant overall, the only significant difference occurred between the WT Control vs KO Stressed.

Supplementary Fig. S2

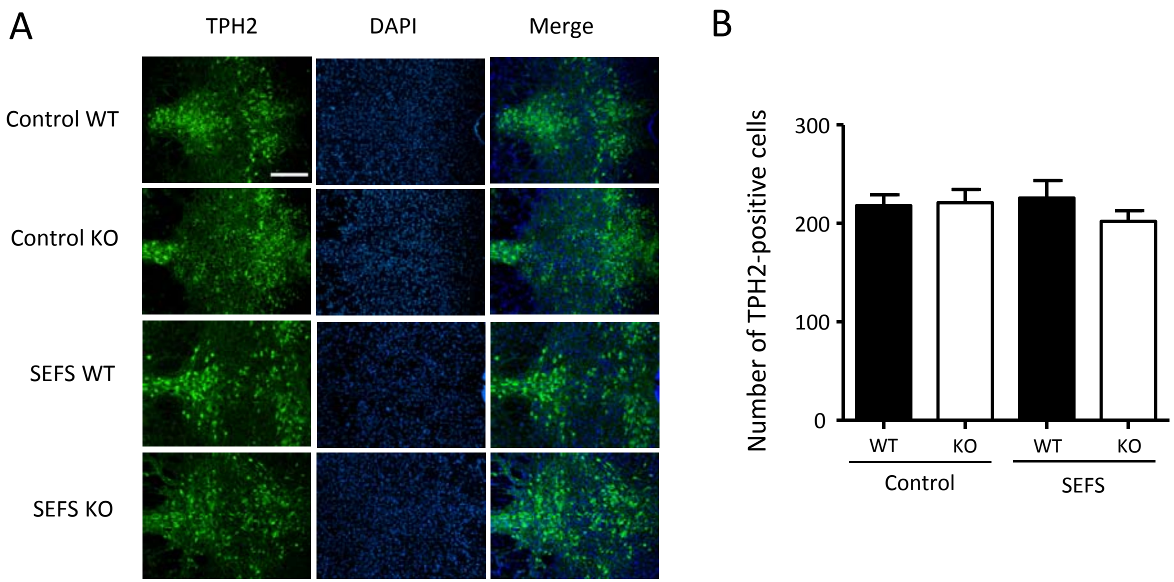

Fig. S3. TPH2 expression in the DRN did not change after a single exposure to forced swimming (SEFS). (A) Representative photomicrographs of TPH2 immunofluorescent staining in the DRN of the WT and MOAP-1−/− mice after a single exposure to forced swimming stress. Scale bar = 200 μm. (B) Number of TPH2 immunopositive cells in the DRN in WT and MOAP-1−/− mice after a single exposure to forced swimming stress, n=3. Data are presented as Mean±SEM. No statistical significance by ANOVA: F=0.741.

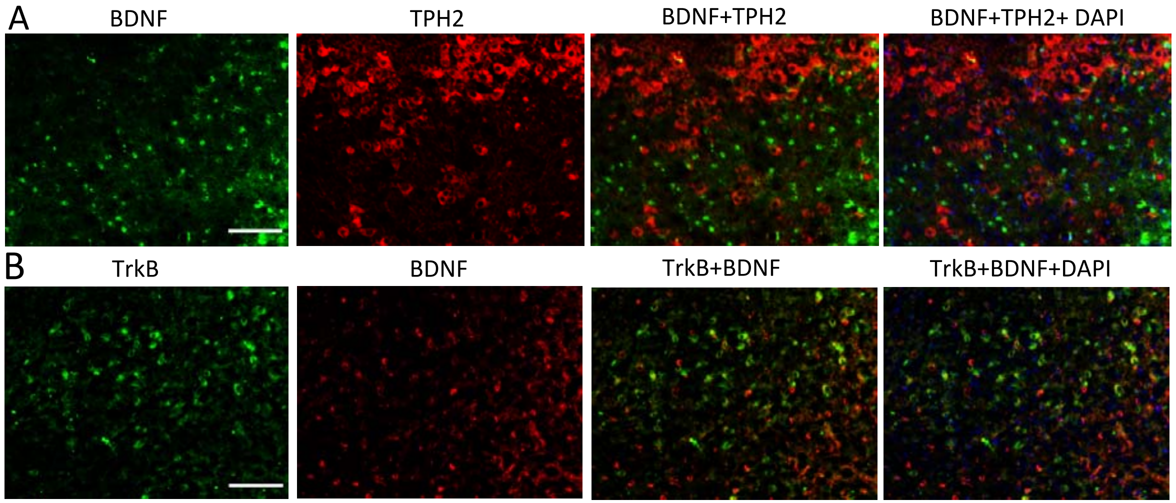

Fig. S4. Double immunostaining of (A) BDNF/TPH2 and (B) BDNF/TrkB receptor in the DRN of wildtype mice. Scale bars = 100 μm. No colocalization was observed.

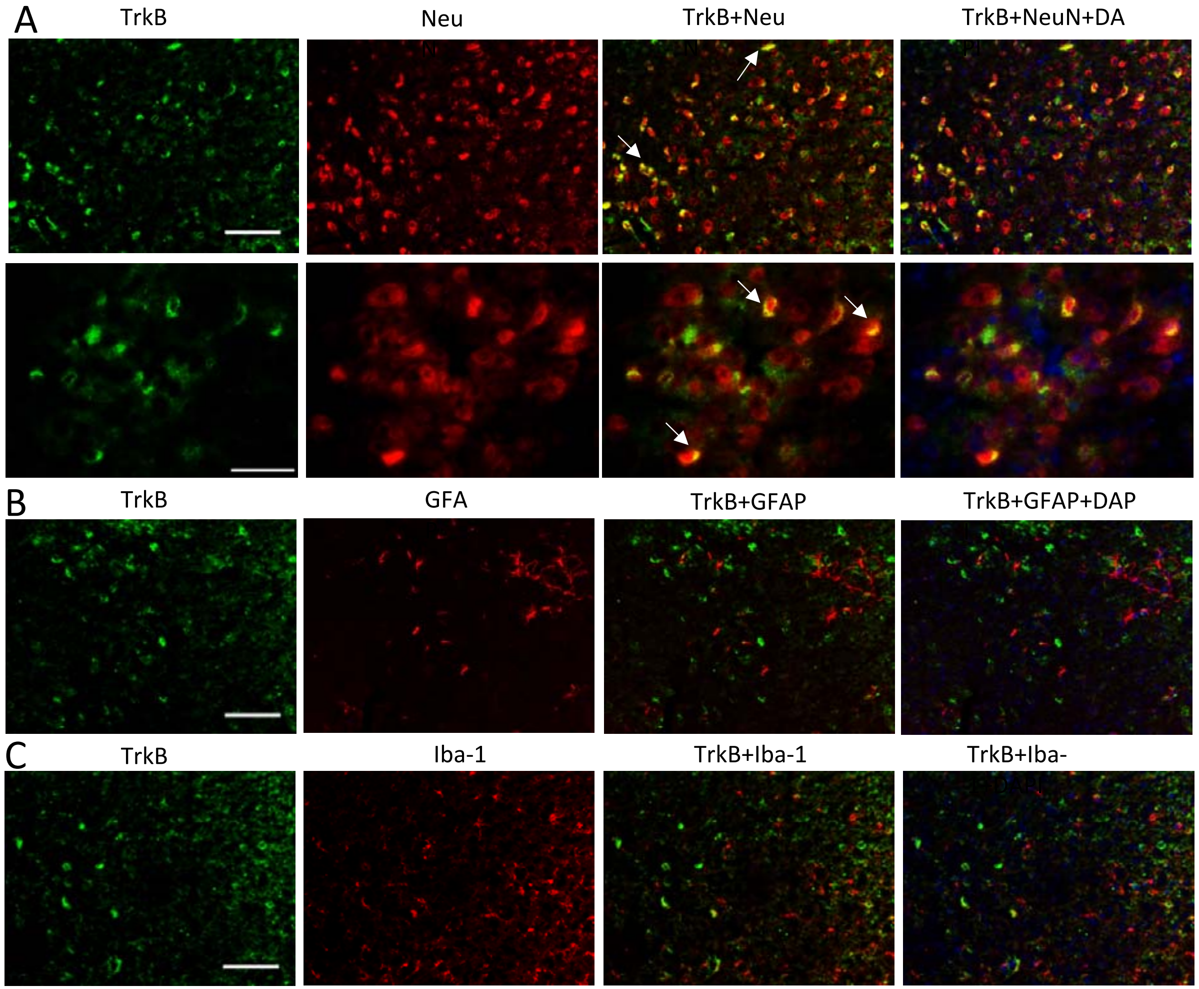

Fig. S5. Cellular localization of TrkB in the DRN of wildtype mice. (A) Double staining of TrkB with NeuN showing colocalization. Scale bar = 100 µm (top) and 50 µm (bottom). White arrows indicate colocalization. (B) Double staining of TrkB with GFAP showing no colocalization. Scale as in A top panel. (C) Double staining of TrkB with Iba-1 showing no colocalization. Scale as in A top panel.
